# The complete mitochondrial genome of the horned lizard *Phrynosoma blainvillii* (Squamata: Phrynosomatidae) from California, USA

**DOI:** 10.1080/23802359.2017.1407706

**Published:** 2017-11-25

**Authors:** Laura Ayala, Ga Hun Boo, Sung Min Boo, Brandon Cluff, Gage H. Dayton, Leah Edwards, Armando Garcia, Mariana Gomez, Thomas A. Jimenez, Jeffery R. Hughey, Timothy Huynh, Natalia I. Marquez, Ana I. Meza, Alexis D. Munoz, Cristina Perez, Dominic Pina, Michael Porras, Paola E. Razo, Alexis Rosales, Bailee Rusconi, Noel Salvador, Alicia Steinhardt, Ruben Tinajero, Nancy Wheat, Frances L. Wong, Ann Wright, Hartnell College Genomics Group

**Affiliations:** a Division of Mathematics, Science and Engineering, Hartnell College, Salinas, CA, USA;; b University Herbarium, University of California, Berkeley, CA, USA;; c Department of Biology, Chungnam National University, Daejeon, Korea;; d Department of Ecology and Evolutionary Biology, University of California, Santa Cruz, CA, USA;; e University of California Natural Reserves, Santa Cruz, CA, USA;; f Department of Molecular, Cell and Developmental Biology, University of California, Santa Cruz, CA, USA

**Keywords:** Horned lizard, mitogenome, *Phrynosoma blainvillii*, Phrynosomatidae, special concern, Squamata

## Abstract

Analysis of *Phrynosoma blainvillii* Gray from Marina, Monterey County, California, using 150 bp paired-end Illumina sequences (Illumina, San Diego, CA) resulted in the assembly of its complete mitogenome. The mitogenome is 16,946 bp in length and contains a putative origin of light strand replication (OL), control region, 22 tRNA, 2 rRNA, and 13 protein-coding genes. Its content and organization are similar to other Squamata. Phylogenetic analysis of *P. blainvillii* resolves it in a clade with *P. sherbrookei* Nieto-Montes de Oca, Arenas-Moreno, Beltrán-Sánchez & Leaché, sister in position to *Uma notata* Baird. Mitochondrial marker analysis of *P. blainvillii* from Marina shows that it belongs to a coastal Santa Lucia Mountain Range haplogroup that is distinct from other populations of *P. blainvillii* in California.

The family Phrynosomatidae consists of North American spiny lizards classified into nine genera and 148 species (Leaché et al. 2015a). Three complete mitogenomes have been published for the family: *Uta stansburiana* Baird & Girard (Leaché et al. 2015b), *Sceloporus occidentalis* Baird & Girard (Kumazawa 2004), and *Urosaurus nigricaudus* Cope (Bernardo et al. 2016). One of the 9 genera, *Phrynosoma* Wiegmann, includes 17 species of horned lizards (Nieto-Montes de Oca et al. 2014). Here, we describe the complete mitogenome of *Phrynosoma blainvillii*, a California Department of Fish and Wildlife ‘Species of Special Concern’, distributed from Northern California to Northern Baja California, Mexico (Leaché et al. 2009).

DNA was extracted from *P. blainvillii* (specimen voucher deposited in the Herpetological Collection – Museum of Vertebrate Zoology at UC Berkeley: MVZ:Herp:283805) collected from the UCSC Fort Ord Natural Reserve, Marina, California using the DNeasy Blood and Tissue Kit (Qiagen, Valencia, CA). The 150 bp paired-end library construction and sequencing was performed by myGenomics, LLC (Alpharetta, GA), yielding 4,978,820 reads. The mitogenome was assembled *de novo* using MEGAHIT (Li et al. 2015) and by mapping the reads against *U. stansburiana* (GenBank NC_027261) with the Medium-Low Sensitivity/Fast setting in Geneious R11 (Biomatters Limited, Auckland, New Zealand). The genes were annotated using MITOS (Bernt et al. 2013) and adjusted in Sequin (https://www.ncbi.nlm.nih.gov/Sequin/index.html). Alignment of the mitogenome to other Squamata was performed with MAFFT (Katoh and Standley 2013). The maximum likelihood analysis was executed using complete mitogenome sequences with T-REX (Boc et al. 2012) and the GTR + gamma model with 1000 fast bootstraps. The tree was visualized with TreeDyn 198.3 at Phylogeny.fr (Dereeper et al. 2008).

The mitogenome of *P. blainvillii* (GenBank MG387969) is 16,946 bp in length and has a base composition of 33.85% A, 26.60% T, 13.07% G, and 26.48% C. It contains 22 tRNA (trnL and trnS are duplicated), two rRNA (rnl, rns), and 13 electron transport and oxidative phosphorylation genes. Eleven of the 13 genes initiate with the ATG start codon, however *cox1* and *nad1* initiate with GTG. Most of the genes terminate with TAA, but *nad2* terminates with TAG, *nad6* with AGG, and *cox1*, *cox2*, and *nad4* with AGA. The *nad6* gene and eight tRNAs encode on the light strand, while the others encode on the heavy strand. The putative OL is located between trnN and trnC, and is 30 bp in length, and the control region is 1540 bp. Phylogenetic analysis of *P. blainvillii* resolves it with *P. sherbrookei* in a fully supported clade sister to the genus *Uma* Baird (Figure 1). This relationship is similar to the findings of previous workers (Reeder and Wiens 1996; Wilgenbusch and de Queiroz 2000). Comparison of the complete *P. blainvillii* mitogenome to published *P. blainvillii* sequences (Leaché et al. 2009) found nearly identical sequences of *nad1* and *nad2* from a specimen from the Santa Lucia Mountain Range, but divergent sequences compared to a specimen of *P. blainvillii* from the nearby Gabilan Mountain Range. These data show that the Fort Ord, Marina and Santa Lucia populations of *P. blainvillii* represent a distinct coastal mitochondrial haplogroup.

**Figure 1.**
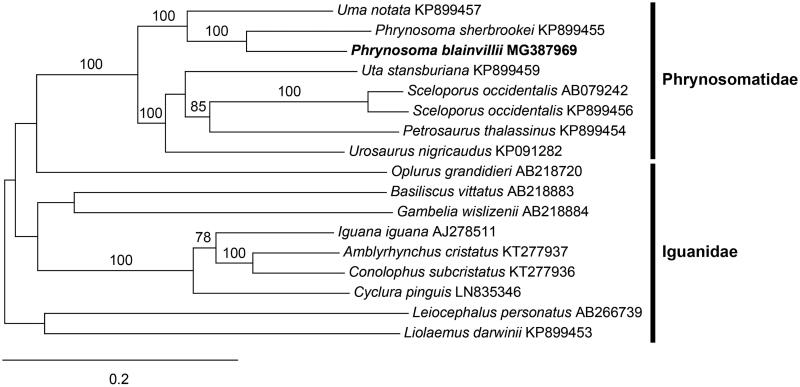
Maximum-likelihood phylogram of *P. blainvillii* and representative Phrynosomatidae and Iguanidae mitogenomes. Numbers along branches are bootstrap supports based on 1000 nreps (<75% support not shown). The legend below represents the scale for nucleotide substitutions.
